# Posttraumatic Growth and Dyadic Adjustment among War Veterans and their Wives

**DOI:** 10.3389/fpsyg.2017.01102

**Published:** 2017-06-30

**Authors:** Yael Lahav, Yaniv Kanat-Maymon, Zahava Solomon

**Affiliations:** ^1^Department of Psychology, University of Southern DenmarkOdense, Denmark; ^2^I-Core Research Center for Mass Trauma, Tel-Aviv UniversityTel-Aviv, Israel; ^3^Interdisciplinary Center HerzliyaHerzliya, Israel; ^4^Bob Shapell School of Social Work, Tel-Aviv University, Tel-AvivNon-US/Non-Canadian, Israel

**Keywords:** posttraumatic growth, dyadic adjustment, posttraumatic stress symptoms, war combat, prisoners of war, secondary traumatization, trauma

## Abstract

The controversy regarding the nature of posttraumatic growth includes two main competing claims: one which argues that posttraumatic growth reflects authentic positive changes and the other which argues that posttraumatic growth reflects illusory defenses. While the former might suggest that posttraumatic growth enhances intimacy and close relationships, the latter might imply that posttraumatic growth hinders interpersonal relations. The present study aimed to test these claims by investigating the association between posttraumatic growth and dyadic adjustment over time at both the individual and dyadic levels, and the potential role of posttraumatic stress symptoms. Former prisoners of war and comparable war veterans and their wives (*n* = 229) were assessed twice, 30–31 (T1) and 35–38 (T2) years after the 1973 Yom Kippur War in Israel, with regard to posttraumatic growth, posttraumatic stress symptoms and dyadic adjustment. Results indicated that posttraumatic growth was associated with both elevated posttraumatic stress symptoms and low dyadic adjustment among both husbands and wives. Posttraumatic stress symptoms at T1 and T2 mediated the association between posttraumatic growth and dyadic adjustment. Wives' posttraumatic growth at T1 predicted posttraumatic growth and dyadic adjustment of the husbands at T2. The higher the wives' posttraumatic growth, the higher the posttraumatic growth and the lower the dyadic adjustment of the husbands in the subsequent measure. The findings suggest that posttraumatic growth reflects defensive beliefs which undermine marital relationships and that posttraumatic growth might be transmitted between spouses and implicated in the deterioration of the marital relationship over time.

## Introduction

Combat and war captivity are highly traumatogenic experiences. Combat exposes the individual to the threat of death and injury. War captivity includes, in addition to combat exposure, prolonged, and deliberate psychological torture aimed at breaking the prisoner' spirit (Herman, 1992). The psychopathological implications of combat and war captivity are not limited to primary trauma survivors, and may be transmitted to their significant others, a phenomenon known as secondary traumatization (e.g., Figley, 1986).

Exposure to a traumatic event might lead to a posttraumatic reaction, most commonly known as posttraumatic stress disorder (PTSD). According to the DSM-IV-TR (American Psychiatric Association, 2000), which the current study relies upon, PTSD symptoms (PTSS) include intrusion (e.g., flashbacks), avoidance (e.g., numbness), and hyper-arousal (e.g., alertness). Research has consistently documented PTSS to be the most common psychiatric consequence of combat and war captivity (Engdahl et al., 1997), and has indicated high rates of PTSS among combatants, former prisoners of war (ex-POWs; Page, 1992), and their spouses (e.g., Galovski and Lyons, 2004), even decades after the traumatic event.

However, it has been suggested that exposure to traumatic events can also result in positive gains or transformations. Most notably, posttraumatic growth (PTG), is defined as the tendency to report enhanced changes in the aftermath of traumatic events in three domains: self-perception, interpersonal relationships, and world view (e.g., Calhoun and Tedeschi, 2006).

Research has documented PTG among individuals who have experienced various traumatic events, both first hand (for reviews see Calhoun and Tedeschi, 2006) and indirectly through intimate interactions with trauma survivors (e.g., Manne et al., 2004). PTG has also been documented following exposure to combat and war captivity in combat veterans (e.g., Aldwin et al., 1994), ex-POWs (e.g., Ursano et al., 1986; Feder et al., 2008), and their wives (e.g., McCormack et al., 2011).

Although PTG has been documented in numerous psychosocial studies, there remains a controversy regarding its nature and long term implications. According to one perspective, PTG is seen as a genuine transformation of basic beliefs about the self and the world, which results from struggling with the effects of trauma (e.g., Calhoun and Tedeschi, 2006). However, according to an alternative perspective, PTG might have an illusory quality that may be maladaptive and hinder coping in the long term (e.g., McFarland and Alvaro, 2000; Lahav et al., 2016). The Janus-face model (Maercker and Zoellner, 2004), suggests that PTG simultaneously includes both a constructive aspect and an illusory aspect. The constructive aspect of PTG is the result of an active struggle with the trauma, and is related to heightened adjustment and well-being, both in the short and long term. However, the effects of the illusory aspect depend on the timing of its usage and the extent of denial involved. Specifically, it is claimed that when the illusory aspect of PTG serves as an acute short term palliative strategy, and co-exists with deliberate thinking about the trauma and the coping effort, it has neither positive nor negative long-term aftereffects. Yet, under conditions in which PTG is solely illusory and serves in the long run as avoidance strategy, strengthening efforts to evade the acknowledgment of the traumatic event and the multiple losses it entails, it has deleterious effects on adjustment. The present study explores reports of PTG decades after the trauma has ended, and not the usage of PTG occuring shortly after the exposure. Hence, drawing upon the Janus-face model also indicates both positive and negative potential implications of PTG, depending on the dominance of the constructive side of PTG and the involvement of denial.

The dispute about the nature of PTG is highlighted by mixed results regarding the association between PTG and PTSS (e.g., Helgeson et al., 2006). While some studies have revealed a negative association between PTG and PTSS (e.g., Frazier et al., 2001), i.e., higher PTG associated with lower PTSS, others have indicated a positive correlation (e.g., Dekel et al., 2012), or a non-significant relationship between the two (e.g., Zoellner and Maercker, 2006).

Although there is ample research on the implications of PTG on the individual's mental health, its consequences with regard to the interpersonal domain, and specifically to the marital relationship, have not been studied. The present study investigates the implications of PTG reported many years after the trauma on dyadic adjustment and the role of PTSS within these associations. In the following sections we will first present the literature regarding dyadic adjustment and PTSS among ex-POWs, combatants and their wives. We will then present perspectives with regard to PTG, dyadic adjustment and the role of PTSS.

A considerable body of research has consistently demonstrated that traumatic events have negative implications on marital relationships. Empirical studies have indicated that for couples in which one partner has experienced combat or war captivity, there have been reports of lower relationship satisfaction and dyadic adjustment (Neria et al., 2000), more communication problems and conflicts (Cook et al., 2004), and more impairments in intimacy and sexual relations (e.g., Zerach et al., 2010).

PTSS is one of the most widely accepted mechanisms underlying the low dyadic adjustment in the aftermath of combat and war captivity (e.g., Cook et al., 2004). For example, intrusive symptoms can amplify preoccupation with the self at the expense of the relationship; avoidance symptoms might lower self-disclosure; and hyperarousal symptoms might amplify interpersonal conflict (e.g., Cook et al., 2004). Research conducted among combat veterans or ex-POWs and their wives indicated that the veterans' PTSS was associated with lower marital satisfaction for both partners (e.g., Cook et al., 2004) and mediate the association between exposure to trauma and low dyadic adjustment among ex-POWs (Dekel et al., 2008). Furthermore, a longitudinal study conducted among fathers in the Army National Guard from a Brigade Combat Team indicated that increases in PTSS over time was associated with poorer dyadic adjustment at a subsequent measurement (Gewirtz et al., 2010).

Despite the fact that PTG refers to the interpersonal domain and also assumes to reflect enhanced changes in relationships, research regarding the implications of PTG on marital relationships is limited. In fact, to the best of our knowledge, former studies that investigated PTG with regard to marital relationships have assessed dyadic adjustment as a predictor of PTG and not as a consequence of growth (e.g., Manne et al., 2004; Weiss, 2004).

Given that the quality of a couple's relationship affects both partners' psychological health (e.g., Proulx et al., 2007), it is important to study the effects of PTG on dyadic adjustment. Furthermore, as PTG by definition is assumed to be linked to enhanced changes in relationships, examining the effects of PTG on the quality of the marital relationship might illuminate the nature of PTG. The present study aimed to fill this gap in the knowledge by investigating the associations between PTG and dyadic adjustment, and the role of PTSS within this association, among war veterans and their wives.

As noted above, the literature implies two main alternatives regarding the implications of PTG in dyadic adjustment, when it is reported long after the trauma ended. The first assumes that if PTG mainly reflects an authentic change it will be linked to strengthened dyadic adjustment (e.g., Maercker and Zoellner, 2004). According to Calhoun and Tedeschi (2006), PTG reflects positive schemas regarding interpersonal interactions. Trauma survivors who experience growth become more emotionally expressive, utilize social supports more than before, and show efforts to improve relationships and greater sensitivity to others (Calhoun and Tedeschi, 2006).

However, according to the alternate view, if, in the long run, PTG serves mainly as an illusory defense that involves denial, it could negatively affect coping with the trauma (e.g., Maercker and Zoellner, 2004) leading to subsequent difficulties in adjustment. These adverse ramifications could, in turn, permeate the individuals' most intimate relationship, resulting in lower dyadic adjustment.

A similar split in the literature arises regarding the potential mediator role of PTSS in the association between PTG and dyadic adjustment. The first alternative assumes that if PTG mainly reflects an authentic transformation, it might ease the survivors' distress and PTSS in the long term (e.g., Maercker and Zoellner, 2004), which, in turn, would enhance dyadic adjustment.

Alternatively, if PTG mainly reflects illusory defenses that serve as a way to deny one's adversity, it might affect PTSS negatively, which in turn undermines dyadic adjustment. Beliefs of growth might act as an avoidance strategy (e.g., Maercker and Zoellner, 2004; Lahav et al., 2017) thereby inhibiting processing the traumatic event and leading to elevated PTSS. This possible negative effect, might, in turn, lead to low dyadic adjustment.

The inter-dependence between spouses is a well-established notion as they affect each other in a multitude of dimensions. Research has consistently demonstrated that veterans' mental health states are implicated in their spouses' well-being (e.g., Renshaw et al., 2011) and the increased distress exhibited by veterans is associated with lower marital satisfaction for both veterans and their partners (e.g., Renshaw et al., 2008). Given that the mental state of the husband and wife are interrelated, one would assume that their PTG and dyadic outcomes would also prove to be interrelated. However, to the best of our knowledge, there has not been a study that has assessed whether PTG and dyadic adjustment in one spouse affects the PTG and dyadic adjustment in the other. The third aim of the present study is to investigate the mutual and reciprocal effects between husbands' and wives' PTG and dyadic adjustment in a longitudinal design.

The present study will examine: (1) the associations between PTG, PTSS, and dyadic adjustment among war veterans and their wives, separately; (2) the mediating role of concurrent, prospective and difference over time in PTSS in the relationships between PTG and dyadic adjustment among war veterans and their wives, separately and; (3) the associations between husbands' and wives' PTG and dyadic adjustment over time.

## Materials and methods

### Procedure and participants

The present study used data from a longitudinal study on the psychological implications of war among veterans and their wives (Greene et al., 2014, for full details). A cohort of Israeli veterans from the 1973 Yom Kippur War and their wives were followed over time. The current study used data which were collected at two points in time: 2003/2004 (T1) and 2008/2011 (T2), from both veterans and their wives.

In order to locate veterans, we used Israel Defense Forces (IDF) files. Wives of veterans were recruited via their spouses. Both husbands and wives were contacted by telephone and asked individually to take part in the study. A battery of questionnaires were administered in their homes or in another location of their choice. Before filling out the questionnaires, participants signed an informed consent form. This study was approved by the Tel Aviv University ethics committee.

#### War veterans

According to records of the Israeli Ministry of Defense, 240 soldiers who served in the IDF land forces were taken prisoner in the 1973 Yom Kippur War. In addition, 280 comparable veterans were sampled from IDF computerized data banks. These individuals participated in the same war, but were not taken captive and were matched on military background and sociodemographics. A total of 227 veterans participated in T1. Of these, 121 were ex-POWs (53.3%) and 106 were combat veterans (46.7%). In T2 there was a total of 294 participants. Of these, 176 were ex-POWs (59.9%) and 118 were combat veterans (40.1%). For all veterans the mean age at T1 was 52.62 (*SD* = 4.56), mean years of schooling was 13.94 (*SD* = 3.46); the majority were secular (61.7%), with an over-average income (35.6%).

#### Veterans' wives

In T1, 213 veterans were married. A total of 165 wives participated in T1. Of these, 90 were ex-POWs' wives (54.5%) and 75 were controls' wives (45.4%). In T2, the number of married veterans increased to 250. Hence, the number of veterans' wives who participated also increased, to 171. Of these, 114 were ex-POWs' wives (66.6%) and 57 were controls' wives (33.1%). The mean age for wives of veterans at T1 was 50.70 (*SD* = 6.36), mean years of schooling was 14.18 (*SD* = 3.18); the majority were secular (57.9%), with an over-average income (35.0%).

In the present study, we used an anchor wherein couples were included in the sample only if both partners participated in at least one wave of measurement. The current sample consisted of 229 couples.

### Handling missing data

Substantial attrition, and in several cases, addition, are very common in longitudinal designs (Collins et al., 2001). In the current study, both of these occurred from T1 to T2. Of the current sample of 229 couples at T1 and T2, respectively: 179 (78.17%) and 187 husbands (81.66%), and 156 (68.12%) and 160 (69.87%) wives had data in regards to the study variables. In addition, data were missing across variables, with some variables having more missing data than others. Hence, we assessed the differences between valid and missing data in each variable and for each spouse and measurement. Overall 19.2–38.9% data were missing across both waves and variables.

To decide whether the data had missing values in a pattern that was random, we conducted analyses of differences between these groups in all of the variables, using Little's Missing Completely at Random (MCAR) test (Collins et al., 2001). The analysis revealed that the data were not missing completely at random, $\chi_{\left(889\right)}^{2}$ = 1021.356, *p* = 0.001. Supplementary analyses revealed that the husbands of wives with missing data regarding PTSS at T2, endorsed significantly lower PTSS symptoms at T1 (*t* = 2.8, *p* = 0.006) and lower PTG at T1 (*t* = 2.1, *p* = 0.04) than husbands of wives without missing data.

As the mechanism of missingness was unknown and there were indications that the missingness was related to the observed data, we assumed that the data were missing at random (MAR). Missing data were handled with maximum likelihood (ML) via the SPSS 22 and AMOS 22 programs. Compared to conventional methods, such as arithmetic mean, listwise or pairwise deletion, ML method was recommended as an optimal method for both attrition and addition of participants over time (Collins et al., 2001). The final sample (after ML was implemented) comprised of 229 Israeli couples (134 ex-POW couples and 95 control couples).

### Measures

#### PTSS (PTSD inventory) (PTSD-I; Solomon et al., 1993)

Husbands' and wives' PTSS was assessed via the PTSD-I, a well-validated, 17-item, self-report questionnaire. The items on the PTSD-I correspond to the DSM-IV-TR diagnosis for PTSD (American Psychiatric Association, 2000). Respondents rated the symptoms experienced in the previous month on a scale ranging from (0) not at all to (4) almost always. Wives' PTSS scores were obtained by asking wives to rate their symptoms related to their husbands' experiences of combat or captivity. The number of positively endorsed symptoms was calculated by counting the items in which the respondents answered “3” or “4.” The PTSD-I has proven psychometric properties and convergent validity (e.g., Solomon et al., 1993). In the present study, Cronbach's alphas were 0.95, 0.96 for husbands and 0.91, 0.91 for wives, at T1 and T2 respectively.

#### Post traumatic growth inventory (PTGI; Tedeschi and Calhoun, 1996)

The PTGI was used to assess the salutary impact of trauma for both husbands and wives. The scale includes 21-items scored on a 4-point scale from (1) I didn't experience this change at all, to (4) I experienced this change to a very great degree. The PTGI includes five subscales, however, in the current study only the total score was calculated. The PTGI has good internal consistency as well as good construct, convergent and discriminant validity (Tedeschi and Calhoun, 1996). In the present study, Cronbach's alphas were 0.94, 0.93 for husbands, and 0.96, 0.94 for wives, at T1 and T2 respectively.

#### Dyadic adjustment scale (DAS; Spanier, 1976)

Husbands' and wives' dyadic adjustment levels were assessed by the DAS which consists of 32 items divided into four subscales: satisfaction, cohesion, consensus, and affection expression. In addition, the total score was computed by summing the ratings on the 32 items. Participants were asked to indicate the extent to which each item describes their current marital relationship. The scale has good convergent and discriminant validity (Heyman et al., 1994). In the current study, Cronbach's alphas were 0.94–0.96 for the total score and 0.90–0.96 for the subscales.

### Data analysis

To assess the associations between PTG, on the one hand, and PTSS and dyadic adjustment, on the other hand, while controlling for study group, partial correlations were conducted. Next, in order to examine whether PTSS at T1 and T2 mediated the link between T1 PTG and T2 dyadic adjustment, we used multiple step mediation (Hayes et al., 2011). Specifically, we examined: (a) whether T1 PTG directly affected dyadic adjustment at T2, controlling for PTSS at T1 and T2; (b) whether T1 PTG indirectly affected dyadic adjustment via PTSS at any of the time points (i.e., T1 and T2, separately); and (c) whether T1 PTG indirectly affected dyadic adjustment via a two-step mediation process (i.e., via PTSS at T1-T2). We controlled for study group as well as for T1 dyadic adjustment in order to take into account the stability of dyadic adjustment over time. To examine whether these indirect paths were significant, we employed accelerated bias-corrected bootstrap analyses.

In order to examine the associations between husbands' and wives' PTG and dyadic adjustment over time, we used an Actor-Partner Independence Model (APIM; Kashy and Kenny, 2000) via AMOS statistics, Version 22. The APIM, “is a model of dyadic relationships that integrates a conceptual view of interdependence in two person relationships with the appropriate statistical techniques for measuring and testing it” (Cook and Kenny, 2005, p. 101). A good model fit to the observed data is suggested if the comparative fit index (CFI), normed fit index (NFI), and the Tucker-Lewis index (TLI) are greater than 0.90 and the root mean square error of approximation (RMSEA) is lower than 0.07.

## Results

### Associations between PTG, PTSS, and dyadic adjustment

Among both spouses, partial correlation analyses indicated that PTG was positively correlated with PTSS beyond the effect of study group. The higher the PTG, the more the PTSS. Among husbands, PTG at T1 was negatively correlated with the levels of dyadic adjustment measures at T2 beyond the effect of study group. Among wives, PTG at T1 and T2 were negatively correlated with the levels of dyadic adjustment measures at T1 and T2 beyond the effect of study group. The higher the PTG, the lower the dyadic adjustment (see Table 1 for husbands' results; see Table 2 for wives' results).

**Table 1:** Partial correlations between the study measures controlling for study group among husbands.

**Measure**	**1**	**2**	**3**	**4**	**5**	**6**	**7**	**8**	**9**	**10**	**11**	**12**	**13**	**14**
1. PTG, T1	–													
2. PTG, T2	0.60^***^	–												
3. PTSS, T1	0.28^***^	0.23^***^	–											
4. PTSS, T2	0.26^***^	0.28^***^	0.72^***^	–										
5. DAS total, T1	−0.06	−0.05	−0.42^***^	−0.38^***^	–									
6. DAS satisfaction, T1	−0.09	−0.07	−0.39^***^	−0.30^**^	0.91^***^	–								
7. DAS cohesion, T1	0.05	−0.04	−0.30^***^	−0.26^**^	0.79^***^	0.65^***^	–							
8. DAS consensus, T1	−0.07	−0.02	−0.40^***^	−0.40^***^	0.79^***^	0.76^***^	0.69^***^	–						
9. DAS affection, T1	−0.06	−0.06	−0.31^***^	−0.35^***^	0.79^***^	0.70^***^	0.54^***^	0.69^***^	–					
10. DAS total, T2	−0.12^**^	−0.02	−0.44^***^	−0.46^***^	0.60^***^	0.59^***^	0.56^***^	0.49^***^	0.39^***^	–				
11. DAS satisfaction, T2	−0.17^**^	−0.07	−0.37^***^	−0.39^***^	0.42^***^	0.47^***^	0.45^***^	0.27^***^	0.28^***^	0.90^***^	–			
12. DAS cohesion, T2	−0.09	−0.09	−0.33^***^	−0.34^***^	0.60^***^	0.53^***^	0.66^***^	0.48^***^	0.42^***^	0.79^***^	0.64^***^	–		
13. DAS consensus, T2	−0.12^*^	0	−0.45^***^	−.46^***^	0.61^***^	0.58^***^	0.50^***^	0.56^***^	0.36^***^	0.94^***^	0.74^***^	0.66^***^	–	
14. DAS affection, T2	−0.15^*^	−0.09	−0.31^***^	−0.37^***^	0.38^***^	0.38^***^	0.26^***^	0.32^***^	0.36^***^	0.76^***^	0.64^***^	0.48^***^	0.73^***^	–
*M* (*SD*)	2.34 (0.67)	2.17 (0.66)	6.96 (5.59)	6.82 (5.69)	106.19 (22.60)	36.70 (7.50)	15.09 (5.45)	46.32 (10.07)	8.08 (2.47)	95.22 (29.57)	31.28 (10.90)	14.08 (5.88)	42.48 (13.57)	7.38 (2.58)
Range	2.76	3	17	20.25	125	40	28.65	57	11	142	51.92	29.72	67.5	12

*PTG, posttraumatic growth; PTSS, posttraumatic symptoms; DAS, dyadic adjustment scale; T1, assessment in 2003; T2, assessment in 2008*.

* *p < 0.05*.

** *p < 0.01*.

*** *p < 0.001*.

**Table 2:** Partial correlations between the study measures controlling for study group among wives.

**Measure**	**1**	**2**	**3**	**4**	**5**	**6**	**7**	**8**	**9**	**10**	**11**	**12**	**13**	**14**
1. PTG, T1	–													
2. PTG, T2	0.78^***^	–												
3. PTSS, T1	0.55^***^	0.46^***^	–											
4. PTSS, T2	0.57^***^	0.52^***^	0.78^***^	–										
5. DAS total, T1	−0.29^***^	−0.18^**^	−0.31^***^	−0.30^***^	–									
6. DAS satisfaction, T1	−0.27^***^	−0.20^**^	−0.29^***^	−0.30^***^	0.90^***^	–								
7. DAS cohesion, T1	−0.13^*^	−0.13^*^	−0.20^***^	−0.17^**^	0.84^***^	0.70^***^	–							
8. DAS consensus, T1	−0.30^***^	−0.16^*^	−0.30^***^	−0.28^***^	0.93^***^	0.74^***^	0.66^***^	–						
9. DAS affection, T1	−0.29^***^	−0.12	−0.32^***^	−0.37^***^	0.81^***^	0.68^***^	0.63^***^	0.74^***^	–					
10. DAS total, T2	−0.35^***^	−0.17^*^	−0.32^**^	−0.33^***^	0.60^***^	0.48^***^	0.36^***^	0.66^***^	0.52^***^	–				
11. DAS satisfaction, T2	−0.37^***^	−0.17^*^	−0.33^***^	−0.27^***^	0.62^***^	0.62^***^	0.32^***^	0.66^***^	0.45^***^	0.83^***^	–			
12. DAS cohesion, T2	−0.18^**^	−0.10	−0.26^***^	−0.24^***^	0.61^***^	0.43^***^	0.56^***^	0.61^***^	0.50^***^	0.71^***^	0.61^***^	–		
13. DAS consensus, T2	−0.30^***^	−0.15^*^	−0.26^***^	−0.29^***^	0.43^***^	0.29^**^	0.22^**^	0.52^***^	0.41^***^	0.91^***^	0.56^***^	0.47^***^	–	
14. DAS affection, T2	−0.30^***^	−0.10	−0.17^**^	−0.31^***^	0.31^***^	0.26^***^	0.19^**^	0.31^**^	0.41^***^	0.78^***^	0.54^***^	0.43^***^	0.74^***^	–
*M (SD)*	2.30 (0.82)	2.24 (0.79)	3.82 (3.80)	4.13 (4.83)	105.69 (24.08)	36.41 (7.39)	15.07 (5.95)	45.98 (11.25)	8.22 (2.49)	103.09 (24.82)	36.07 (7.95)	14.82 (5.33)	44.60 (13.33)	7.60 (2.75)
Range	12.00	68.79	25.00	40.00	136.99	12.40	65.00	35.00	45.50	141.50	18.82	16.72	3.95	3.20

*PTG, posttraumatic growth; PTSS, posttraumatic symptoms; DAS, dyadic adjustment scale; T1, assessment in 2004; T2, assessment in 2011*.

* *p < 0.05*.

** *p < 0.01*.

*** *p < 0.001*.

### Multistep mediation of PTSS in the relation between PTG and dyadic adjustment

**Figure 1:**
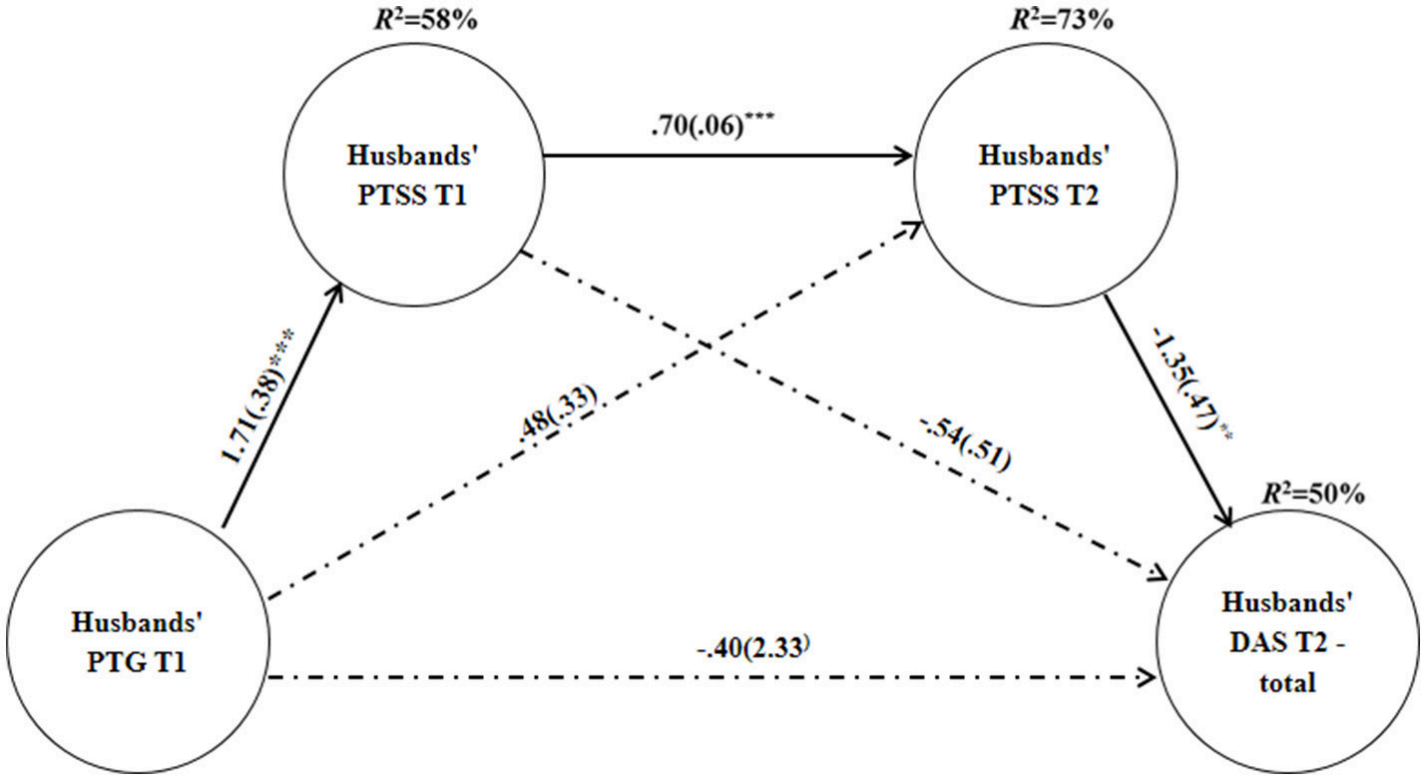
Unstandardized coefficients b(SE) for the association between PTG and Dyadic Adjustment total score through the sequential mediation of PTSS at T1 and T2, among Husbands. Explained variance is located above all dependent variables. PTG, posttraumatic growth; PTSS, posttraumatic symptoms; DAS, dyadic adjustment scale. ^**^p < 0.01, ^***^p < 0.001.

Multiple step mediation analyses among the husbands (Table 3) revealed that PTG had a non-significant direct effect for dyadic adjustment, except for the cohesion subscale. PTG indirectly predicted the husbands' dyadic adjustment via PTSS at T2 as well as via both PTSS at T1 and T2 for the dyadic adjustment total score and all subscales (See Figure 1 for dyadic adjustment total score). Higher PTG at T1 predicted higher PTSS at T1 (*B* = 1.71, *SE* = 0.38, *P* < 0.001), which, in turn, increased the levels of PTSS between T1 and T2 (*B* = 0.70, *SE* = 0.06, *P* < 0.001). Next, higher levels of PTSS at T2 were associated with low dyadic adjustment at T2 (*B* = −1.35, *SE* = 0.47, *P* = 0.004; *B* = −0.54, *SE* = 0.19, *P* = 0.005; *B* = −0.23, *SE* = 0.09, *P* = 0.02; *B* = −0.53, *SE* = 0.22, *P* = 0.02; *B* = −0.13, *SE* = 0.05, *P* = 0.009, for dyadic adjustment total score, satisfaction, cohesion, consensus, and affection, respectively).

**Table 3:** Bootstrap 95% confidence intervals for predicting dyadic adjustment by PTG through PTSS in time 1 and time 2 among husbands and wives.

**Measure**	**DAS total**	**DAS satisfaction**	**DAS cohesion**	**DAS consensus**	**DAS affection**
**HUSBANDS**					
Direct	{−4.9985, 4.2098}	{−2.9297, 0.8421}	{0.1477, 1.9502}^*^	{−2.2557, 2.1195}	{−0.7100, 0.2442}
Indirect through T1 PTSS	{−3.4385, 1.2416}	{−1.0888, 0.7898}	{−0.5781, 0.2043}	{−2.0401, 0.1158}	{−0.2873, 0.1876}
Indirect through T2 PTSS	{−1.9182, −0.0041}^*^	{−.7487, −0.0047}^*^	{−0.03053, −0.0071}^*^	{−0.8828, −0.0050}^*^	{−0.1910, −0.0007}^*^
Indirect through T1 and T2 PTSS	{−3.4463, −0.2788}^*^	{−1.4567, −0.1656}^*^	{−0.7128, −0.1098}^*^	{1.5546, −0.0741}^*^	{−0.3794, −0.0382}^*^
**WIVES**					
Direct	{−8.6758, −0.6303}^*^	{−3.3917, −0.8863}^*^	{−1.0249, 0.7738}	{−4.0650, 0.6634}	{−1.1532, −0.1497}^*^
Indirect through T1 PTSS	{−3.0300, 2.9537}	{−1.8815, 0.1247}	{−0.9924, 0.3929}	{−0.9851, 2.1684}	{−0.1602, 0.8889}
Indirect through T2 PTSS	{−1.9934, 0.7487}	{−0.1375, 0.7452}	{−0.4428, 0.2028}	{−1.4728, 0.1533}	{−0.4323, −0.0526}^*^
Indirect through T1 and T2 PTSS	{−3.0390, 1.2190}	{−0.2423, 1.1903}	{−0.7246, 0.3427}	{−2.4080, 0.2991}	{−0.6632, −0.0902}^*^

*95% Confidence Intervals are presented in brackets. Confidence intervals that do not include 0 (null association) are significant*.

* *Significant at 0.05. PTG, posttraumatic growth; PTSS, posttraumatic symptoms; DAS, dyadic adjustment scale*.

**Figure 2:**
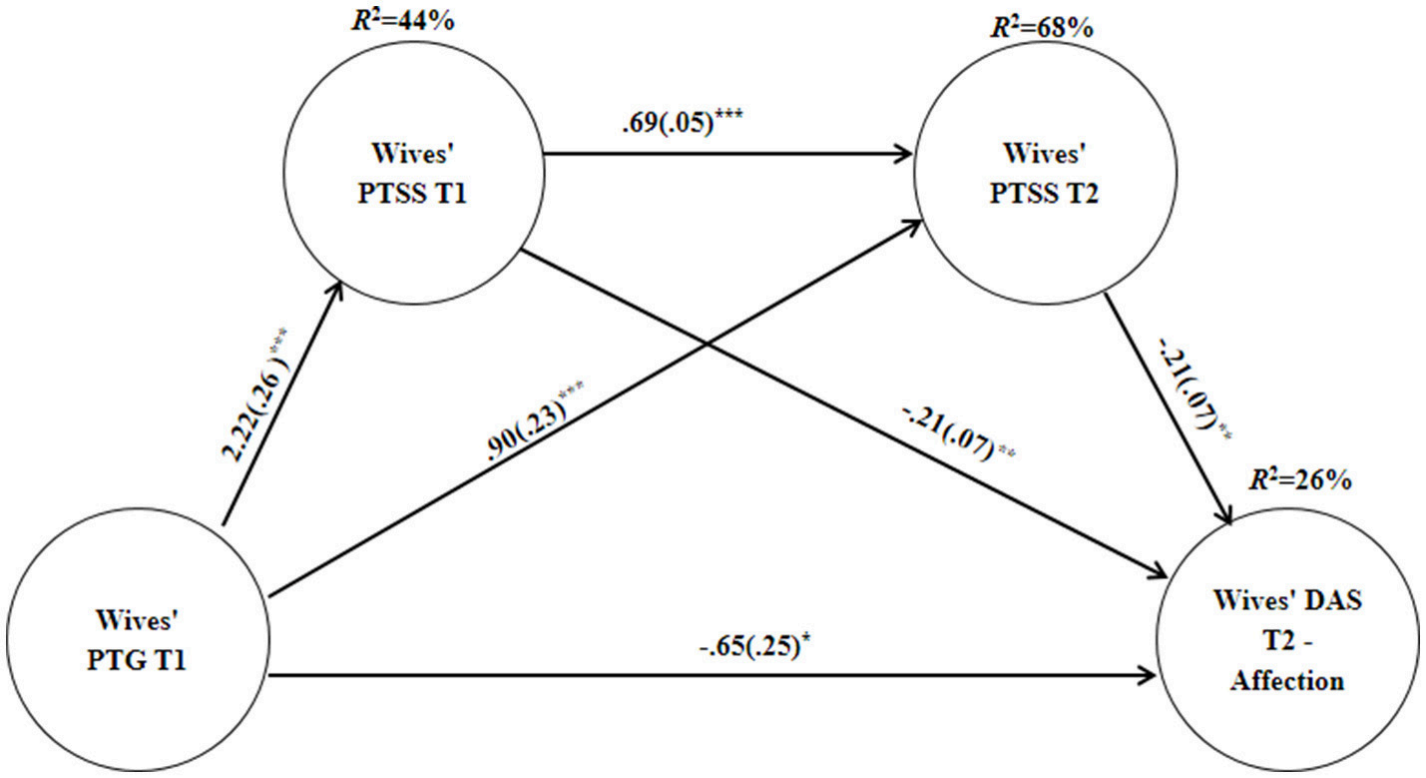
Unstandardized coefficients b(SE) for the association between PTG and Dyadic Adjustment affection subscale through the sequential mediation of PTSS at Tl and T2, among Wives. Explained variance is located above all dependent variables. PTG, posttraumatic growth; PTSS, posttraumatic symptoms; DAS, dyadic adjustment scale. ^*^p < 0.05, ^**^p < 0.01, ^***^p < 0.001.

Analyses among the wives revealed (Table 3) that PTG had a significant direct effect on dyadic adjustment, except for the cohesion and consensus subscales. PTG did not have an indirect effect on dyadic adjustment, apart from the affection subscale (See, Figure 2). PTG indirectly predicted the wives' affection via PTSS at T2 as well as via both PTSS at T1 and T2. Higher PTG at T1 predicted higher PTSS at T1 (*B* = 2.22, *SE* = 0.26, *P* < 0.001), which, in turn, increased the levels of PTSS between T1 and T2 (*B* = 0.69, *SE* = 0.05, *P* < 0.001). Next, higher levels of PTSS at T2 were associated with low affection at T2 (*B* = −0.21, *SE* = 0.07, *P* = 0.003).

### APIM model for wives' and husbands' PTG and dyadic adjustment

The fit indices of the APIM model indicated that the theoretical model was a good representation of the data, χ^2^_(6)_ = 10.21, *p* = 0.116, χ^2^*/df* = 1.701, CFI = 0.98, NFI = 0.97, TLI = 0.90, RMSEA = 0.06. We wanted to estimate a simpler and parsimonious model, containing only the significant paths found. We compared its fit indices to the general model, arguing for many paths of impact. Non-significant difference of the two chi-squares suggest that the omission of the non-significant parameters did not reduce model and indicate favors to the simpler model (Ledermann et al., 2011).

No significant difference was found between the two chi-squares, Δχ^2^_(9)_ = 7.125, *p* = 0.624. Hence, we proceeded with the more parsimonious simple model omitting non-significant paths. Fit indices of the simpler model indicated that the model was an excellent representation of the data, χ^2^_(15)_ = 17.335, *p* = 0.299, χ^2^*/df* = 1.155, CFI = 0.99, NFI = 0.94, TLI = 0.98, RMSEA = 0.03.

**Figure 3:**
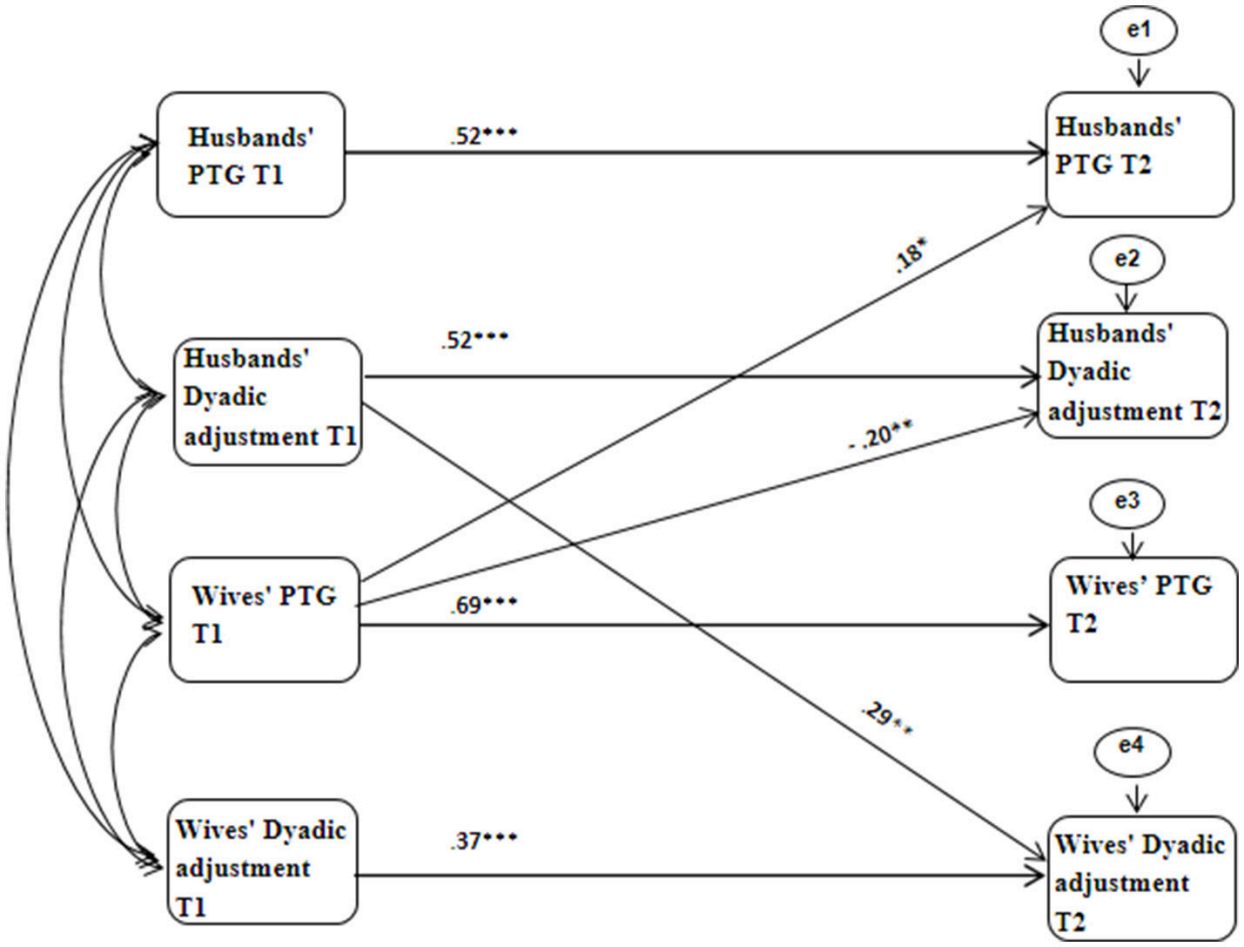
APIM nested Model of Husbands' and Wives' PTG and dyadic adjustment. Curved lines represent covariates between constructs. Solid lines represent significant predictions. The other prediction axes were non-significant. ^*^p < 0.05, ^**^p < 0.01, ^***^p < 0.001.

Figure 3 and Table 4 display the standardized coefficients and significant paths for the parsimonious nested model. The analysis revealed high stability of PTG and dyadic adjustment over time among both partners. Those with high levels of PTG and dyadic adjustment at T1 tended to have high levels of PTG or dyadic adjustment at T2. More importantly, the analysis revealed that the initial level of wives' PTG at T1 predicted husbands' dyadic adjustment at T2, above and beyond the stability of husbands' dyadic adjustment. The higher the wives' PTG at T1, the lower the husbands' dyadic adjustment at T2. The analysis also revealed that the initial level of wives' PTG at T1 predicted husbands' PTG at T2, above and beyond the stability of husbands' PTG. The higher the wives' PTG at T1, the higher the husbands' PTG at T2. Lastly, the analysis revealed that the initial level of husbands' dyadic adjustment at T1 predicted wives' dyadic adjustment at T2, above and beyond the stability of wives' dyadic adjustment. The higher the husbands' dyadic adjustment at T1, the higher the wives' dyadic adjustment at T2. The other prediction axes were non-significant.

**Table 4:** APIM nested model for the associations between husbands' and wives' PTG and DAS over time—standardized coefficients.

**Path**	**β**	**SE**
PTG T1 (H) → PTG T2 (H)	0.52^***^	0.07
DAS T1 (H) → DAS T2 (H)	0.52^***^	0.09
PTG T1 (W) → PTG T2 (W)	0.69^***^	0.07
DAS T1 (W) → DAS T2 (W)	0.37^***^	0.10
PTG T1 (W) → PTG T2 (H)	0.18^*^	0.06
DAS T1 (H) → DAS T2 (W)	0.29^**^	0.11
PTG T1 (W) → DAS T1 (H)	−0.20^**^	2.53

*PTG, posttraumatic growth; DAS, dyadic adjustment scale, (H), husbands; (W), wives*.

* *p < 0.05*.

** *p < 0.01*.

*** *p < 0.001*.

We explored the possibility that the reported findings change as a function of participants' study group (i.e., ex-POWs and their wives vs. war veterans and their wives). To that end, we conducted multigroup APIM models that estimated the relation between husbands' and wives' PTG and dyadic adjustment, separately for each group. The multigroup model did not fit the data well, χ^2^_(29)_ = 57.57, *p* = 0.001, χ^2^*/df* = 1.99, CFI = 0.87, NFI = 0.81, TLI = 0.69, RMSEA = 0.07. Furthermore, the chi-square difference test (Brown et al., 1990) yielded non-significant values, Δχ^2^_(4)_ = 3.411, *p* = 0.492, suggesting that the results do not change as a function of study group.

## Discussion

The current study explored PTG in the context of marital relationships on both the individual and dyadic levels. The present results revealed that among husbands who were exposed directly to combat or war captivity, as well as their wives, who were exposed indirectly to these traumatic events, PTG was not only associated with elevated PTSS but also low dyadic adjustment. PTSS mediated the association between PTG and all of the dyadic adjustment subscales among husbands, and the association between PTG and the dyadic adjustment affection subscale among wives. Lastly, the wives' PTG predicted higher PTG and lower dyadic adjustment among the husbands over time.

The current findings were inconsistent with previous findings that indicated PTG to be related to enhanced adjustment, manifested in lower PTSS (e.g., Frazier et al., 2001; Carver and Antoni, 2004). It could be argued that the implications of PTG depends upon the severity of the trauma; however, in this study PTG was not found to alleviate subsequent distress for either the ex-POW or control dyads. Alternatively, it might be that the discrepancy between findings is rooted in the measurement of PTG. While this study used the PTGI (Tedeschi and Calhoun, 1996), a validated and widely-used questionnaire, previous studies which indicated PTG to be adaptive, predicting lower subsequent distress, often used unstandardized measures of growth (Dekel et al., 2012).

The current findings were consistent with evidence indicating a positive relationship between self-reported PTG and PTSS (e.g., Helgeson et al., 2006; Zalta et al., 2017). Moreover, our findings demonstrated, for the first time, that PTG is not only related to the intensification of PTSS over time, but also to negative changes in dyadic adjustment. This pattern of results might suggest that PTG reflects illusory defenses (e.g., Lahav et al., 2016) and are in line with former studies that indicated a gap between reports of PTG and actual interpersonal behavior among trauma survivors (Hobfoll et al., 2007). Hence, while trauma survivors report growth, they may suffer from the deterioration of their most intimate relationship.

Holding beliefs of growth might reflect the efforts of primary and secondary trauma survivors to cope with trauma's negative repercussions. Individuals who have been exposed to severe traumatic events experience a dismantling of basic beliefs about the self and the world and, as a result, suffer from intense feelings of horror and helplessness (Janoff-Bulman, 1989). While facing extreme distress they might find possessing growth beliefs to be comforting and adopt these beliefs as a coping strategy. However, our findings suggest that in the long term this method of coping could be maladaptive and might be associated with low dyadic adjustment.

One possible explanation for the association between PTG and lowered dyadic adjustment is that maintaining positive beliefs of growth might limit the trauma survivors' ability to authentically express emotions and receive support within the marital relationship. While trying to cling on to the growth beliefs, war veterans and their wives might avoid expressing their vulnerabilities and sharing their difficulties regarding their trauma. Emotions such as helplessness, hopelessness, frustration, anger, accusation, and blame regarding their traumatic past are not pronounced within the relationship and the current difficulties, resulting from the traumatic experience, are not genuinely expressed. Moreover, in order to keep the defensive beliefs of growth, war veterans and their wives might avoid asking their spouses for help, further lowering the chance of receiving the much needed social support from within their relationship. These tendencies might impair intimacy and satisfaction in marital relationships (Swann et al., 1994), thereby undermining the dyadic adjustment. It should be noted that the current explanation is speculative in nature, as the present study did not assess the previously mentioned mechanisms, such as wives' emotional authenticity or support within the marriage. Future prospective studies should explore the processes underlying the relations between PTG and dyadic adjustment.

At the same time, as the current results indicated, PTG might negatively impact dyadic adjustment through increased PTSS. Trauma survivors, such as ex-POWs, war veterans and their wives, who maintain growth beliefs, may use it as a way to deny the negative consequences of the trauma. Under these conditions, PTG could serve as a way to avoid acknowledging their emotional pain and losses resulting from the trauma, which prevents working through the traumatic event (Maercker and Zoellner, 2004) and modifying the fear structure that is responsible for posttraumatic reaction (Foa et al., 1989). This, in turn, enhances distress and PTSS over time, which negatively affects marital relations and thus lower dyadic adjustment (Cook et al., 2004).

The present results indicated, however, that the aforementioned path, is more evident among husbands than wives. While PTSS mediated the association between PTG and all of the dyadic adjustment subscales among husbands, it mediated only the association between PTG and the dyadic adjustment affection subscale among wives. One possibility is that the results reflect gender differences. Another possibility is that the results are rooted in the type of exposure to trauma. While the negative effect of PTG on dyadic adjustment is potentially explained mainly by the amplification of PTSS among primary trauma survivors, this might be apparent only in part among secondary trauma survivors. Lastly, it might be that the differences in the magnitude of posttraumatic reactions are responsible for the results. Hence, PTSS has a broader role as a mechanism among the husbands, who suffer from elevated PTSS, compared to the wives, who reported lower levels. The present study does not allow for differentiation between these alternatives, therefore we offer only speculations.

Our results regarding the prediction of the husbands' PTG by the wives' PTG are the first of their kind. The current findings further the notion of transmission between spouses in the aftermath of trauma. It seems that the transmission within the marital dyad is not restricted to posttraumatic reaction, as indicated in *the contagion theory* (Figley, 1986), but may also occur in regards to posttraumatic growth beliefs.

Interestingly, growth cognitions were found to be transmitted from the wives to the husbands, and not the other way around. It could be that these findings reflect the important role of the wives in shaping the way their traumatized husbands cope. As men tend to rely primarily on their wives for intimacy (e.g., Hobfoll et al., 1996), they might adopt their wives' growth beliefs. Specifically, one may speculate that interacting with a wife who maintains growth beliefs could enhance the husband's risk of implementing defensive growth beliefs as an avoidant strategy.

Although the transmission of growth cognitions within the marital dyad perhaps enhances the existence of a similar outlook regarding the traumatic event, at the same time it could be related to negative outcomes and specifically to lower dyadic adjustment. Investigation of these associations between PTG and dyadic adjustment among both spouses indicated that wives' PTG predicted a decrease in the husbands' dyadic adjustment over time, but not the other way around. One might offer two main explanations for the present trend.

The directionality of the associations between spouses might be rooted in gender roles. It is possible that both men and women with high PTG not only avoid expressing their own vulnerabilities, they are also defensive in the face of others' emotional distress, and experience it as a threat toward their growth beliefs. However, while women often have close relationships with other women, men tend to be more dependent on their wives for emotional support (e.g., Hobfoll et al., 1996). Therefore, men might feel more helpless in the face of their spouses' emotional defensiveness, compared to women, and report lower dyadic adjustment as a consequence of their wives' PTG.

Alternatively, the present findings might be related to the different types of exposure to trauma. Primary trauma survivors, as the husbands in our study, exposed particularly to manmade trauma, often suffer from elevated shame and self-blame regarding the traumatic event (Leskela et al., 2002) and are sensitive to others' responses regarding their trauma (Hong et al., 2011). Moreover, due to elevated emotional distress, firsthand trauma survivors often tend to be more dependent on their significant others for emotional support (Dekel, 2007). Hence, it might be that trauma survivors who were exposed directly to trauma, such as ex-POWs and war combatants, are more vulnerable to their spouses' growth beliefs, compared to their wives, who were exposed indirectly. Ex-POWs and war combatants might feel invalidated or ashamed of their difficulties when facing their wives' growth beliefs regarding the trauma, and respond with heightened aloofness and emotional detachment in the relationship. These, in turn, sabotage their relationship, leading to low dyadic adjustment.

In our data, gender and type of trauma exposure are intertwined. Hence, we were unable to distinguish between these competing explanations regarding the directionality of the associations between spouses. Moreover, the effects of gender role and type of trauma exposure in association with PTG and dyadic adjustment have not been studied. Therefore, no definite conclusions can be reached.

Several limitations may have affected our findings. First, this study was based on self-report measures, which may be subject to response biases and shared method variance. Second, the present study did not include data regarding PTG and dyadic adjustment immediately after the trauma, rather only decades after the traumatic event. This presented us with major constraints in our ability to assess whether the ramifications of PTG on dyadic adjustment depended on the amount of time it is in effect. Third, the current study did not include data regarding PTG or PTSS in relation to other potential traumatic events that the participants might have experienced or data regarding the suggested mechanism for the relations between PTG and dyadic adjustment. This prevented us from controlling for the effects of other important variables that may have shaped the present results. Lastly, as was previously mentioned, gender and type of trauma exposure were intertwined in the present data. This prevented us from distinguishing between their different effects. Future prospective studies should use PTG measures at different time intervals to assess the temporal effects of PTG on dyadic adjustment, and consider gender as well as type of trauma exposure separately.

The present findings have important implications for theory and treatment of direct and indirect survivors of trauma. Our results call attention to the possible role of PTG with regard to negative marital outcomes. War veterans, ex-POWs and their wives who report PTG might be at-risk for low dyadic adjustment. Moreover, this potential negative effect of PTG on the marital realm may be transmitted from one spouse to the other. This possibility suggests the need for caution by the therapist when treating trauma survivors in regards to encouraging reports of PTG—although in some cases these reports might reflect true positive changes, in others they might indicate efforts to deny the trauma and could have negative implications. However, given the controversy regarding the nature of PTG, we recommend that more studies be conducted before any further conclusions can be drawn.

## Author contributions

YL and YK Made substantial contributions to the conception and design of the work as well as the analysis and interpretation of data for the work; Drafted the work and revised it critically for content; Gave final approval of the version to be published; Agreed to be accountable for all aspects of the work in ensuring that questions related to the accuracy or integrity of any part of the work are appropriately investigated and resolved. ZS Made substantial contributions to the conception and design of the work as well as the analysis and interpretation of data for the work; Drafted the work and revised it critically for content; Gave final approval of the version to be published.

### Conflict of interest statement

The authors declare that the research was conducted in the absence of any commercial or financial relationships that could be construed as a potential conflict of interest.
